# Supplementary material for: Histopathological and Immunological Findings in the Common Marmoset Following Exposure to Aerosolized SARS-CoV-2
Source: Viruses. 2022 Jul 21;14(7):1580. doi: 10.3390/v14071580 (PMC9322862; doi:10.3390/v14071580)
Supplement: Supplementary file 1 [file viruses-14-01580-s001.zip › viruses-1766436-supplementary.pdf]

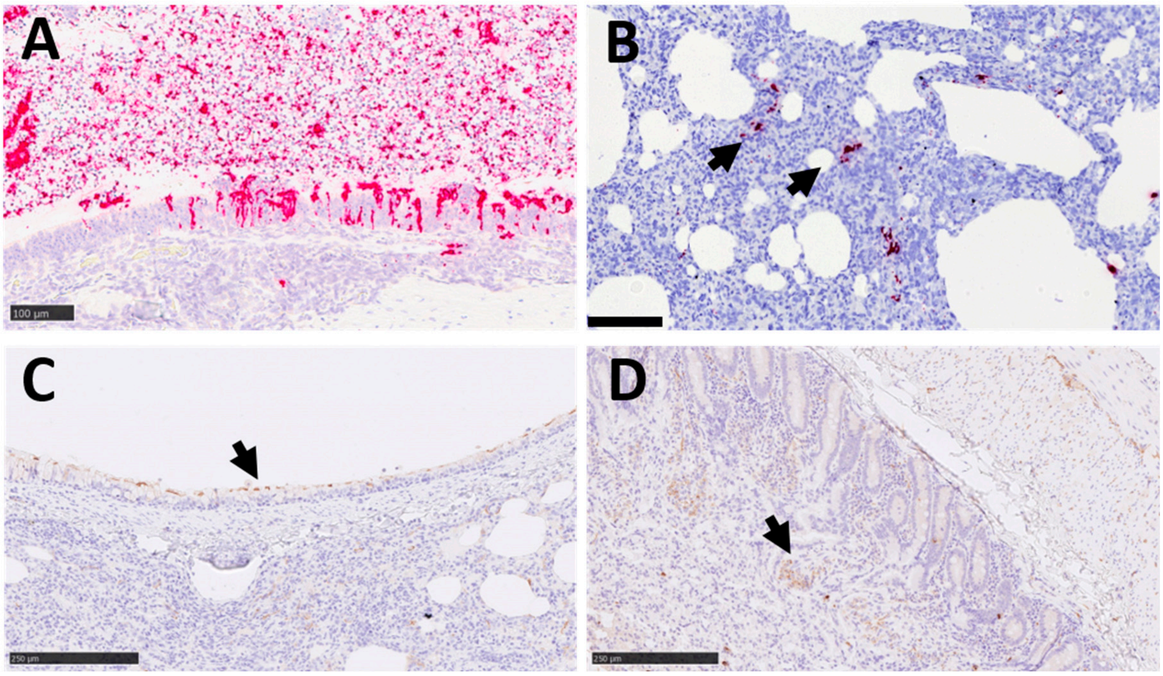

Figure S1. Positive control images from animals infected with SARS-CoV-2. RNAScope ISH stain of virus RNA (*S-gene*) in hamster nasal cavity lung showing intense positive staining in the epithelium and exudates (**A**) and mild positive staining in rhesus macaque lung showing multiple positive cells (arrows) within areas of interstitial pneumonia (**B**); ACE2 IHC stain of a large bronchus from rhesus macaque showing moderate positive reaction in the bronchial epithelium (arrow) (**C**); TMPRSS2 IHC detection in small intestine mucosa (arrow) and other layers from rhesus macaque (Bar = 250 μm) (**D**).
